# Early prediction of spontaneous Patent Ductus Arteriosus (PDA) closure and PDA-associated outcomes: a prospective cohort investigation

**DOI:** 10.1186/s12887-019-1708-z

**Published:** 2019-09-13

**Authors:** Jonathan L. Slaughter, Clifford L. Cua, Jennifer L. Notestine, Brian K. Rivera, Laura Marzec, Erinn M. Hade, Nathalie L. Maitre, Mark A. Klebanoff, Megan Ilgenfritz, Vi T. Le, Dennis J. Lewandowski, Carl H. Backes

**Affiliations:** 1 Center for Perinatal Research, Abigail Wexner Research Institute at Nationwide Children’s Hospital, Columbus, Ohio USA; 2 Department of Pediatrics, Nationwide Children’s Hospital, 700 Children’s Way, Columbus, Ohio 43205 USA; 3 Division of Epidemiology, College of Public Health, The Ohio State University, Columbus, Ohio USA; 4 The Heart Center, Nationwide Children’s Hospital, Columbus, Ohio USA; 5 Department of Biomedical Informatics, Center for Biostatistics, The Ohio State University, Columbus, Ohio USA; 6 Department of Obstetrics and Gynecology, College of Medicine, The Ohio State University, Columbus, Ohio USA

**Keywords:** patent ductus arteriosus, preterm infant, echocardiogram, prospective cohort, prediction modeling

## Abstract

**Background:**

Patent ductus arteriosus (PDA), the most commonly diagnosed cardiovascular condition in preterm infants, is associated with increased mortality and harmful long-term outcomes (chronic lung disease, neurodevelopmental delay). Although pharmacologic and/or interventional treatments to close PDA likely benefit some infants, widespread routine treatment of all preterm infants with PDA may not improve outcomes. Most PDAs close spontaneously by 44-weeks postmenstrual age; treatment is increasingly controversial, varying markedly between institutions and providers. Because treatment detriments may outweigh benefits, especially in infants destined for early, spontaneous PDA closure, the relevant unanswered clinical question is not whether to treat all preterm infants with PDA, but whom to treat (and when). Clinicians cannot currently predict in the first month which infants are at highest risk for persistent PDA, nor which combination of clinical risk factors, echocardiographic measurements, and biomarkers best predict PDA-associated harm.

**Methods:**

Prospective cohort of untreated infants with PDA (n=450) will be used to predict spontaneous ductal closure timing. Clinical measures, serum (brain natriuretic peptide, N-terminal pro-brain natriuretic peptide) and urine (neutrophil gelatinase-associated lipocalin, heart-type fatty acid-binding protein) biomarkers, and echocardiographic variables collected during each of first 4 postnatal weeks will be analyzed to identify those associated with long-term impairment. Myocardial deformation imaging and tissue Doppler imaging, innovative echocardiographic techniques, will facilitate quantitative evaluation of myocardial performance. Aim1 will estimate probability of spontaneous PDA closure and predict timing of ductal closure using echocardiographic, biomarker, and clinical predictors. Aim2 will specify which echocardiographic predictors and biomarkers are associated with mortality and respiratory illness severity at 36-weeks postmenstrual age. Aim3 will identify which echocardiographic predictors and biomarkers are associated with 22 to 26-month neurodevelopmental delay. Models will be validated in a separate cohort of infants (n=225) enrolled subsequent to primary study cohort.

**Discussion:**

The current study will make significant contributions to scientific knowledge and effective PDA management. Study results will reduce unnecessary and harmful overtreatment of infants with a high probability of early spontaneous PDA closure and facilitate development of outcomes-focused trials to examine effectiveness of PDA closure in “high-risk” infants most likely to receive benefit.

**Trial registration:**

ClinicalTrials.gov NCT03782610. Registered 20 December 2018.

**Supplementary Information:**

**Supplementary Information** accompanies this paper at 10.1186/s12887-019-1708-z.

## Background

Patent ductus arteriosus (PDA), the most commonly diagnosed cardiovascular condition in preterm infants, is associated with an eight-fold increase in mortality [1] and multiple, harmful longer-term outcomes including bronchopulmonary dysplasia (BPD) [2–4], intestinal injury [5, 6], brain damage [3, 7–9], cerebral volume loss [10, 11], and congestive heart failure [12, 13]. PDA results in an enduring blood flow in preterm infants between the aorta and the pulmonary artery. The ductus, an essential component of fetal circulation, normally closes shortly after birth in term infants, but remains open (patent) at one-month of age in approximately 70% of <30-weeks gestation preterm infants [12, 14]. PDA-associated symptoms including mesenteric, renal, and cerebral hypoperfusion and pulmonary edema secondary to pulmonary overcirculation develop in 60% of those with persistent PDA [11, 12, 15].

Despite association with worsened outcomes in preterm infants, PDA closure has become increasingly controversial among cardiologists and neonatologists [16], with treatment strategies varying markedly between institutions [17–19] and individual providers [20]. Within the first postnatal month, the majority of neonatal providers still routinely administer nonsteroidal anti-inflammatory drugs, or less commonly acetaminophen, to at least some infants with PDA with the expectation of increased early ductal closure [18, 20]. Because the majority of PDAs in preterm infants close spontaneously by 44-weeks postmenstrual age (PMA) when left untreated, the frequencies of both early medication treatment and surgical or catheter-based closure ligation for persistent PDA have declined over the last decade [17–20]. Given a lack of validated evidence to identify which subset of preterm infants is most likely to benefit from PDA closure [3], the decision to treat PDA remains subjective. Randomized clinical trials [3, 21, 22] and innovative observational studies [18, 23] have not demonstrated net improvement in death, BPD, or neurodevelopmental impairment following routine medical or interventional treatments (surgical ligation, catheter-based closure) of all preterm infants with PDA. However, the effectiveness of more selective PDA treatment closure strategies remains unclear [3, 12, 13].

Nonsteroidal anti-inflammatory drug treatment is linked to nephrotoxicity [24], acute renal failure [25], decreased cerebral and intestinal blood flow [26, 27], intestinal perforation when co-administered with glucocorticoids [28–30], and failure to close the PDA in one-third of infants [31], and costs between $1458 to $1875 per three-dose treatment course [32, 33]. Acetaminophen, a less studied medical option, is associated with neurologic impairment [34, 35]. Surgical ligation via thoracotomy requires exposure to general anesthesia [36, 37]. Vocal-cord paralysis and post-operative hemodynamic instability are also potential risks [38–40]. PDA closure via heart catheterization is potentially less-invasive [41–44]; however, it remains understudied and carries potential complications including arterial injury [41–43]. Since all forms of PDA closure are expensive and associated with adverse effects [38, 41–43, 45, 46], treatment harms may outweigh benefits, especially for the majority of infants destined for early, spontaneous PDA closure.

Still, treatment of some infants with symptomatic PDA is necessary [13]. A subset of preterm infants with persistent PDA continues to display PDA-associated symptoms, including volume overloading of their immature heart and lungs, and worsening respiratory failure [3, 13, 47]. Chronic PDA exposure is associated with congestive heart failure [13, 48, 49] and death [1, 3, 50].

The American Academy of Pediatrics and experts on PDA in preterm infants agree that future outcomes-based randomized clinical trials are needed to determine the effectiveness of selective pharmacological and/or interventional treatments for “high-risk” infants with PDA [3, 12, 13, 51]. The goal is to eventually deliver prompt, personalized PDA treatment to only those infants most likely to benefit [3, 52, 53], thereby reducing the side-effects and costs associated with unnecessary and potentially harmful PDA overtreatment. Unfortunately, no validated prediction models currently exist to permit early identification of those infants with increased probabilities of persistent PDA and PDA-associated harm. As a first step in trial design, the American Academy of Pediatrics has called for the development of comprehensive, echocardiographic- and biomarker-based PDA risk-stratification tools [3].

The objectives of this study are to use a prospective cohort of untreated preterm infants with PDA to accurately predict the timing of spontaneous ductal closure (and conversely, long-term ductal patency) in preterm infants with PDA, and the identification of measurable echocardiographic predictors and biomarkers that are present in the first postnatal month that are associated with mortality, chronic lung disease (CLD), and long-term neurological impairment. These results will ultimately improve neonatal patient care by informing the design of outcomes-focused randomized clinical trials that will examine the effectiveness and timing of PDA closure in those “high-risk” infants most likely to receive benefit [41].

## Methods

### Research design overview

A number of clinical [54], echocardiographic [4, 55–57], and biomarker [58] variables have known associations with an increased likelihood of either spontaneous PDA closure or PDA-associated morbidity [3]. However, no one has incorporated all reported predictors to develop comprehensive, clinically-focused PDA prediction models. In addition, most prior investigations have been limited by retrospective study designs [4], with echocardiographic surveillance at the clinician’s discretion rather than uniform echocardiogram collection at pre-specified time points. The few prospective studies were limited by exposure measurements at single, early time points [57, 58], short durations of PDA exposure [57], and/or small sample sizes [57, 58]. Historically, most PDA investigations have been conducted at sites favoring early, aggressive PDA treatment, thus preventing an unbiased examination of the relationships between early PDA indicators, spontaneous closure, and outcomes following persistent PDA [4]. The proposed research is innovative because we will prospectively collect sequential echocardiographic measures, serum and urine biomarkers, and important clinical risk factors within a cohort of untreated infants with PDA. We will utilize that data to develop robust models to evaluate their combined ability to predict both spontaneous PDA closure timing, and PDA-associated mortality, respiratory, and long-term neurodevelopmental outcomes. This study uses a prospective cohort and a rigorously designed observational study to answer proposed questions that cannot be answered with a randomized trial, but that are crucial to future PDA trial development.

### Study subjects

We will recruit a cohort of <30-weeks gestation preterm infants consecutively enrolled using the following inclusion and exclusion criteria:

#### Inclusion criteria

1. Infants born between 23-weeks + 0 days (23^0/7^ weeks) and 29^6/7^ weeks of gestation, inclusive
2. Admitted to a study network neonatal intensive care unit (NICU) within 72-hours of birth
3. PDA noted on initial screening echocardiogram at <72 postnatal hours

#### Exclusion criteria

1. Life-threatening congenital abnormalities including congenital heart disease (other than PDA or small atrial septal defects/patent foramen ovale/muscular ventriculoseptal defects)
2. Infants whose parents have chosen to allow natural death (do not resuscitate order)

### Recruitment

#### Patient screening for eligibility and recruitment

Cohort entry will occur within 72 hours after birth, a time of PDA patency in >95% of infants born at <30-weeks gestational age [54]. The research coordinator will evaluate new admissions; written informed consent will be obtained by a study nurse or co-investigator. Inpatient recruitment for the *primary study cohort* (n=450) is ongoing (start date 04/01/19; first patient enrolled (04/02/19) with a goal of 2 years. Because prediction models should be validated in a population separate from that in which they were created, inpatient recruitment of a *validation cohort* (n=225) will begin immediately following primary study cohort enrollment completion. Infants will remain in their respective cohort until follow-up at 22 to 26-months corrected age.

#### Recruitment sites

Recruitment will take place within the Nationwide Children’s Hospital (NCH) Neonatal Network, one of the largest neonatal intensive care networks in the United States. The NCH Neonatal Network is comprised of 9 hospitals in central Ohio within a 20-minute drive from the NCH research campus, has 268 neonatal beds, and had >420 admissions of infants born at <30-weeks gestation in 2016. Patient demographics, including sex and race/ethnicity, are largely representative of the US population. All sites share clinical-practice guidelines, are staffed by NCH neonatologists, and electronically transmit echocardiographic images to NCH for cardiologist interpretation.

The NCH Neonatal Network is committed to a guideline-driven approach for the care of infants born at <30-weeks gestation [59]. The NCH Neonatal Network designed and implemented a regional consensus guideline for PDA management in 2012 (Slaughter and Backes, unpublished). Local guidelines mandate that no pharmacologic therapy be used to treat PDA. Interventions to close the PDA (surgical or catheter-based closure) may only be considered after 30 postnatal days as a “last resort” for patients with severe respiratory illness, signs of PDA-associated systemic hypoperfusion (e.g., metabolic acidosis, hypotension, oliguria) and echocardiographic findings of >1.5 mm diameter PDA with increased left cardiac volume load, maximum ductal velocity >2 m/s, or decreased, absent, or reversed end-diastolic abdominal aortic flow. The PDA consensus guidelines and minimally interventional PDA treatment strategies provide a unique opportunity to examine determinants of spontaneous PDA closure among extremely preterm infants who did not receive therapy [20]. Consistent with the switch to guidelines, from 2013-2016 NCH did not perform any surgical PDA ligations or catheter-based closures prior to 30 days postnatal. Since 2012, only 2.6% of infants with PDA have received catheter-based closure, at a median of 38.1 weeks PMA [25^th^-75^th^ percentiles: 35.4 - 41.4 weeks].

### Data Collection

#### Data entry and monitoring

Patient demographic, clinical, and sample data will be collected in REDCap (Research Electronic Database Capture), a robust, secure web-based electronic capture tool that allows for real-time data entry with embedded logic and range checks, skip patterns, and missing data alerts to ensure quality control, while minimizing the amount of missing data, and assigned a study ID number [60]. Study data collection forms were designed and implemented with the support of The Ohio State University Center for Clinical and Translational Science, and are stored behind the NCH firewall. The study research coordinator will enter data for each enrolled preterm infant, including clinical, echocardiographic, and biomarker data. Study statisticians will monitor data for completeness and accuracy.

#### Clinical signs

The clinical cardiac examination will be performed consistent with standards outlined by the American Heart Association and American College of Cardiology [61]. To ensure competency and reliability, training in performance and documentation of the cardiac examination will be ensured prior to study commencement by a pediatric cardiologist who will assess inter- and intra-rater reliability. To ensure standardization of the cardiac examination, the pediatric cardiologist will also perform the standardized exam on 10-20% of enrolled infants on the same day as the research nurse exam and provide feedback or correction as needed. All examiners will be masked to echocardiographic and biomarker results.

#### Echocardiographic measures

All echocardiograms will be performed by dedicated and trained research sonographers, including the baseline echocardiogram <72 hours postnatal to determine study eligibility. Consistent with American Heart Association and American College of Cardiology standards [62], the sonographers will have formal training and be certified in pediatric echocardiography. Infants with evidence of a PDA on the baseline echocardiogram will undergo weekly echocardiograms for the first 4 weeks, then biweekly echocardiograms until PDA closure or NICU discharge; an echocardiogram will also be completed at 36-weeks PMA per study protocol. If the baseline echocardiogram shows no evidence of a PDA, infants will continue to be followed prospectively, but without additional echocardiographic studies. If discharged home with an open ductus, infants will follow-up with the pediatric cardiologist (*per NCH Neonatal Network standard of care*) in the outpatient neonatal-cardiology clinic. All echocardiograms will be performed with a dedicated research ultrasound (CX50; Philips, Amsterdam, The Netherlands). Images will be obtained with the narrowest sector angle to maximize frame rate (goal frame rate >100 frames/s) for optimal image quality [63, 64]. Images will be saved and interpreted by a single pediatric cardiologist who will be masked to patient and clinical data. All recordings will be measured in triplicate and averaged [65]. Consistent with previous work [65–70], images will be analyzed using a dedicated workstation (Tomtec USA, Chicago, IL, USA) [71].

### Risk measurement

During the first postnatal month, variables collected will allow for adequate control of confounding due to severity of illness, as the independent value of echocardiographic measurements and laboratory biomarkers in predicting important outcomes is established (Aims 2 and 3). Early severity of illness may be both a predictor of the duration of PDA patency and a modifier of the effect of echocardiographic and biomarker-derived PDA measurements on PDA closure (Aim 1).

### Clinical measures

#### Predictor variable data collection within the first postnatal month

In addition to echocardiograms and laboratory-measured biomarkers, we will collect multiple clinical variables during the perinatal and neonatal period (postnatal days 0-28) to facilitate predicting the time of spontaneous PDA closure (**Aim 1**), and to control for confounding within our models for mortality and 36-weeks PMA respiratory outcomes (**Aim 2**) and 22 to 26-months neurodevelopmental outcomes (**Aim 3**). The following data will be collected: 1) patient demographics; 2) antenatal risk factors; 3) early postnatal measures of illness severity; 4) diagnoses that present in the first postnatal month (neonatal period); 5) medications; and 6) physiologic measures (Table 1). An overview of the study timeline for each patient is shown in Table 2.

**Table 1:** Clinical predictor variables

Patient Demographics	Antenatal Risk Factors	Early Postnatal Illness Severity	Diagnoses in Early Postnatal Period^a^	Medications (by date, dose^b^, route)	Physiologic Measures (date)
Birth GA Birth weight z-score for GA Sex Race/Ethnicity Transported from outside birth hospital Singleton or multiple gestation Social status (BSMSS)	Maternal corticosteroid administration Maternal magnesium sulfate administration Pre-eclampsia Clinical and histological chorioamnionitis	Apgar scores Score for Neonatal Acute Physiology (SNAPPE-II) variables [72] Variables from NRN Extremely Preterm Outcomes Prediction Tool [73, 74]	IVH Grade 3 or 4 (only included in models after routine ultrasound at 7 postnatal days) Necrotizing enterocolitis Pneumothorax Spontaneous intestinal perforation Seizure	Indomethacin Caffeine Diuretics Inhaled corticosteroids Surfactant treatment Vitamin A Total daily fluid intake^b^	Daily respiratory support modality Mean daily FiO_2_ Oxygen saturation index Mean arterial blood pressure^b^ Daily urine output^b^

^a^ Will only include diagnoses in models that were present prior to model specific week during postnatal weeks 1 to 4

^b^ Will weight adjust by kg

*GA* gestational age, *BSMSS* Barratt Simplified Measure of Social Status, *IVH* intraventricular hemorrhage

**Table 2:** Patient study timeline

	Study Period								
	<72 h	Week 1	Week 2	Week 3	Week 4	Bi-weekly	36-weeks PMA	Every 2-3 months ^f^	22 to 26-months CA
Enrollment									
Eligibility screen (Echocardiogram)	X								
Informed consent	X								
Data collection									
Biomarkers		X	X	X	X				
Echocardiography ^a^		X	X	X	X	X ^d^			
Assessments									
Respiratory support							X		
Echocardiography ^a^							X ^e^	X	X
Bayley III ^b^									X
Catheter-based PDA Closure ^c^									X

^a^ traditional, myocardial deformation imaging, tissue Doppler imaging

^b^ Bayley Scales of Infant and Toddler Development, 3^rd^ Edition (Gross Motor Development Scaled Standard Score postnatal age, Fine Motor Development Scaled Standard Score postnatal age, Cognitive Composite Score, Language Composite Score)

^c^ patients with persistent PDA at 22 to 26-months corrected age (CA)

^d^ obtained bi-weekly until 36-weeks postmenstrual age (PMA) if PDA remains open

^e^ All infants receive echocardiogram at 36 weeks irrespective of previous PDA status

^f^ until documented ductal closure per local standard of care

Note: If PDA closed, additional weekly echocardiograms not obtained

#### Clinical markers associated with mortality and neurodevelopmental impairment

We will include variables routinely collected in preterm infants within the first 12-hours postnatal that comprise the Score for Neonatal Acute Physiology with Perinatal Extension-II (birth weight, 5-minute Apgar score, small [<10^th^ percentile] for gestational age, lowest mean blood pressure, lowest temperature, pO_2_/FIO_2_, lowest serum pH, presence of multiple seizures, and low urine output) [72]. This score is a validated predictor [75] of mortality [72, 76], CLD of prematurity [77], and long-term neurodevelopmental impairment risk [78, 79]. We will also collect *Eunice Kennedy Shriver* National Institute of Child Health and Human Development (NICHD) Neonatal Research Network (NRN) *Extremely Preterm Outcomes Prediction Tool* variables [73]. These variables (e.g., antenatal corticosteroid exposure, female sex, singleton birth, and higher birth weight) are associated with a reduced risk of death and/or neurodevelopmental impairment [74].

#### Clinical markers associated with 36-weeks PMA respiratory status

To determine which early echocardiographic parameters and biomarkers in preterm infants with PDA are independently predictive of respiratory illness severity at 36-weeks PMA (**Aim 2**), early risk variables from the best clinical prediction model for bronchopulmonary dysplasia (BPD) severity (NICHD NRN Neonatal BPD Outcome Estimator) will be included [80, 81]. Preterm birth-associated CLD in infants at 36-weeks PMA is secondary to BPD, a consequence of preterm lung exposure to the extra-uterine environment. Inflammation from alveolar, systemic, and vascular causes including PDA contribute to disease severity [82–85]. These variables include (but are not limited to): 1) gestational age; 2) birth weight; 3) race/ethnicity; 4) sex; 5) respiratory support (none, nasal cannula, continuous positive airway pressure, conventional ventilation, or high-frequency ventilation); and 6) fraction of inspired oxygen (FiO_2_). Pulse oximetry continually measures peripheral capillary oxygen saturation throughout NICU hospitalization as standard of care. We will incorporate the daily oxygen saturation index (mean airway pressure × FiO_2_ × 100 ÷ peripheral capillary oxygen saturation), which has been validated as a non-invasive alternative to oxygenation index for assessing neonatal hypoxic respiratory failure severity [86].

#### Gestational age as a predictor of spontaneous PDA closure

**Fig. 1:**
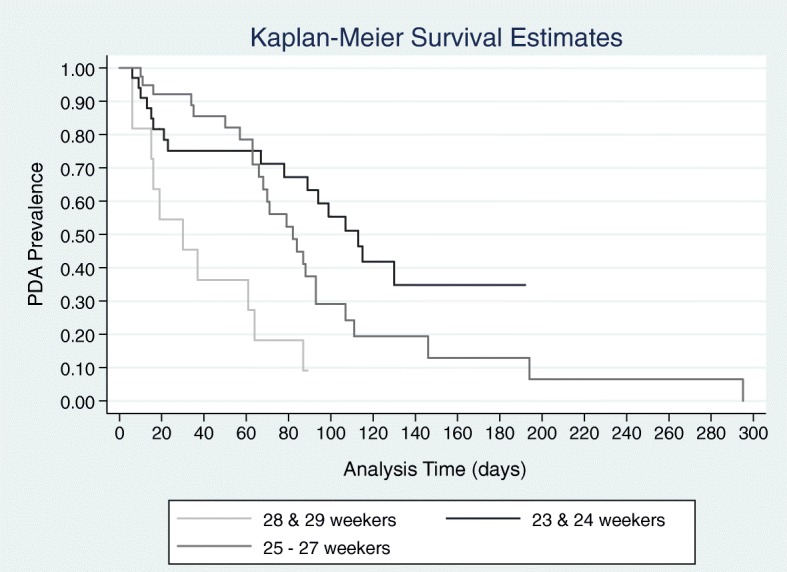
Inpatient Ductal Patency by Gestation over Time. 50% patent ductus arteriosus (PDA) prevalence in preterm infants (n=244): 23-24 weeks (113 d); 25-27 weeks (82 d); 28-29 weeks (30 d). Log-rank test, p=0.0003

We retrospectively evaluated (10/2012–7/2017) local infants with echocardiography-diagnosed PDA who were born 23 to 30-weeks gestation and who had at least 2 echocardiograms to allow PDA status verification (open/closed) over time (n=244) (Fig. 1). We evaluated gestational age, birth weight, sex, race, and 5-minute Apgar scores using a multivariable Cox regression. Gestational age was a significant predictor of ductus closure (Hazard ratio: 1.38, 95% confidence interval: 1.20, 1.59). Given our culture of PDA non-treatment in central Ohio relative to most US institutions, the proportion of patients receiving ≥2 echocardiograms was small. Nevertheless, our finding of a higher prevalence of ductal patency in lower gestational age infants, is strikingly similar to findings reported in a larger cohort (HR: 1.28, 95% confidence interval: 1.20, 1.36) [54].

#### Effect modification by indomethacin for intraventricular hemorrhage

A 3-day prophylactic course of indomethacin may be initiated on the date of birth (postnatal days 0-2) to reduce the frequency of intraventricular hemorrhage. Since indomethacin prophylaxis may contribute to PDA closure in some infants by modifying the effect of determined predictors on PDA-associated outcomes [87], we will evaluate for an interaction when creating prediction models. Persistent ductal patency following prophylaxis may also be a marker of delayed PDA closure. Similar to PDA treatment, the frequency of indomethacin prophylaxis varies between institutions and individual providers [20, 88]. Our retrospective data (10/2012–7/2017) showed that a small minority (15.7%) of clinicians within the NCH Neonatal Network treat infants born ≤29-weeks gestation with indomethacin prophylaxis.

### Prospective collection of PDA indicators in untreated infants

#### Traditional indicators

The clinical signs associated with PDA shunting depend on shunt magnitude and the compensatory ability of the immature myocardium to handle the additional volume load [89]. Historically, some NICUs relied solely on clinical examination for PDA diagnosis [90]. While more recently, investigators have challenged the isolated use of clinical signs [90–99], no studies have prospectively evaluated the accuracy and reliability of clinical examination in a large, prospective cohort of <30-weeks gestation infants, or evaluated those findings alongside echocardiographic or biomarker data.

Traditional echocardiography is the gold-standard for PDA diagnosis and provides assessment of ductal diameter, shunting pattern, and volume [100]. Echocardiographic indices can be used to define the hemodynamic significance of PDA. However, because the relative benefits and limitations of PDA echocardiographic indexes remain poorly characterized [100], we will comprehensively examine a multitude of potential parameters. All traditional echocardiograms will assess standard M-mode, two-dimensional, pulsed-wave Doppler, continuous-wave Doppler, and color Doppler evaluations (PDA size [46, 52, 101–104], shunt pattern, shunt volume [55, 100, 105]).

#### Advanced indicators

##### Tissue Doppler imaging

Tissue Doppler imaging can provide quantitative measures of the velocity of myocardial contraction and relaxation throughout the cardiac cycle as a reflection of cardiac performance. Because both systolic and diastolic information are contained in a single tracing, simultaneous isovolemic contraction and relaxation time intervals can be obtained. Tissue Doppler imaging can also be used to calculate a myocardial performance index that reflects both systolic and diastolic global function [106]. In older patients, quantitative assessment of cardiac function with tissue Doppler imaging is a more sensitive indicator of myocardial dysfunction and may provide earlier detection prior to the more qualitative changes observable with traditional echocardiography [107]. While tissue Doppler imaging measurements have been published for both children [108] and fetuses [109], minimal data are available on infants with pathology. Our group was one of the first to show that both collection and interpretation of tissue Doppler imaging are feasible in extremely preterm infants [65].

Tissue Doppler images will be obtained from the apical 4-chamber view with the point of interest along the annulus at the following 3 segments: right ventricle free wall; interventricular septum; and left ventricle free wall. Tissue Doppler imaging tracings will demonstrate a systolic wave, early and late diastolic waves for each segment, and simultaneous isovolemic contraction and relaxation time intervals.

##### Myocardial deformation imaging

Myocardial deformation imaging assesses strain (change in myocardium length relative to its resting length, expressed as a percentage) and strain rate (deformation over time) [110–112]. In contrast with traditional echo, but similar to tissue Doppler imaging, myocardial deformation imaging quantitatively assesses myocardial function [63, 113–118]. However, unlike tissue Doppler imaging, myocardial deformation imaging is not influenced by loading conditions (preload or afterload), which may be highly variable in premature infants, particularly those requiring intensive care [65, 119].

Right and left ventricle myocardial deformation imaging strain will be assessed using two-dimensional, apical 4-chamber images [110, 113]. The myocardial walls will be divided into 6 segments. Strain curves will be traced from the basal lateral atrioventricular valve annulus to apex to basal interventricular septum for both right and left ventricles. Strain curves will only be accepted after visual inspection of tracing with manual adjustment of range of interest. If tracing is not adequate in all 6 segments per visual inspection and the software, the image will not be used [63].

##### Biomarkers

If either the clinical exam or biomarkers can replace the more costly and labor-intensive echocardiographic techniques at specific postnatal time-points in some predictive models (each echocardiogram is >10-times the cost of the proposed biomarker tests), health care costs would be greatly reduced [120]. Interest in biomarkers to diagnose, stage, and predict hemodynamic and cardiovascular disease in adults and children continues to increase [100, 121–123]. However, no studies have determined, in a large prospective cohort of extremely low birth weight infants, the accuracy and reliability of serum and urinary biomarkers to predict PDA duration and associated outcomes, nor have those findings been evaluated alongside clinical and echocardiographic data*.* The selected biomarkers appear uninfluenced by gestational age, birth weight, and antenatal and postnatal events, making them ideal for evaluating PDA-related myocardial damage and hemodynamic status in preterm infants [120, 124–136].

Weekly blood and urine samples for biomarker analysis will be collected within one calendar day of the echocardiogram for the first 4 weeks postnatal. Blood draws (0.5 mL) will be collected concurrently with weekly nutrition labs that are obtained on all premature infants per local standard of care. Blood samples will be capillary (always concomitant with scheduled clinical blood draws) or arterial if a pre-existing indwelling line is present. Cotton balls will be put in the diapers of enrolled infants to collect urine. De-identified and labeled blood and urine samples will be transported on ice to the Center for Perinatal Research at NCH where samples will be centrifuged, and frozen in a dedicated freezer at −70°C for batch analysis. We have locally validated the serum and urine biomarkers for our specific patient population.

##### Serum biomarkers

To avoid excessive blood collection in preterm infants two serum natriuretic peptide biomarkers were selected a priori: beta-natriuretic peptide and N-terminal pro-B-type natriuretic peptide, which have been shown to predict likelihood of spontaneous PDA closure, respiratory outcomes, mortality, and neurodevelopment [124–129, 131, 137–140]. Both are cardiac hormones that rapidly respond to volume and pressure overload and are elevated in patients with left and right heart failure. Concentrations of both peptides directly correlate with severity of ductal shunting [133, 134]; levels decrease with successful ductal closure [120, 125, 131, 141]. Brain natriuretic peptide is significantly related to shunt magnitude at the time of measurement, and has good discriminating power for detecting moderate-to-large PDA shunts [125].

##### Urinary biomarkers

Ductal shunting may decrease renal and mesenteric bed perfusion [40, 142, 143]. We will use urine (non-invasive, minimal processing) to evaluate two exploratory biomarkers of end-organ ischemia. Neutrophil gelatinase-associated lipocalin is a well-described diagnostic marker of acute renal failure [144–147] with high sensitivity and specificity [148–152]. Heart-type fatty acid-binding protein is an intracellular lipid-binding protein, and expression is stimulated by lipid metabolism alterations, including ischemia. Increased heart-type fatty acid-binding protein levels suggest mesenteric hypoperfusion [153]. Small pilot studies have shown that urinary measurement of both peptides is feasible in preterm infants and appear promising as tools for quantifying the effect of a PDA on systemic perfusion [15, 154].

### Outcome measures

#### Aim 1

To estimate the probability of spontaneous PDA closure and predict the timing of ductal closure using echocardiographic, biomarker, and clinical predictors obtained within the first postnatal month.

Until documented PDA closure, echodardiograms will be conducted weekly for the first 4 weeks postnatal and biweekly thereafter, between study entry and 36-weeks PMA on all inpatient study participants. Based on local pilot data, we expect 15% of PDAs (90% small, <1.5 mm; 10% moderate, 1.5-2 mm) to remain patent at NICU discharge. All preterm infants with PDA at discharge will follow-up with the pediatric cartiologist at the dedicated neonatal-cardiology clinic within The Heart Center at NCH (sole pediatric cardiology provider-site for central Ohio). Per local standard of care, these infants will receive echocardiograms at 2-3 month intervals until documented closure; any infants with a persistent PDA at 22 to 26-months postnatal age (~5% of those discharged with a persistent ductus) will undergo catheter-based closure to prevent long-term complications of PDA (Table 2) [48, 49]. This will facilitate precise documentation of ductal patency duration from 72-hours postnatal until neurodevelopmental follow-up at 22 to 26-months. We will model clinical measures, weekly biomarker measurements, and weekly echocardiography-derived predictors (as described above) from the first 4 postnatal weeks, when the decision to initiate pharmacologic treatment closure for PDA is most commonly made [18]. The *primary outcome* for Aim 1 is documented PDA closure by 36-weeks PMA.

##### Expected Outcomes

A combination of echocardiographic measures, biomarkers, illness severity, and patient demographics is expected to accurately predict in the first postnatal month, both the probability of an infant’s PDA closing and the duration of PDA patency. Developing a clinically useful prediction model will allow clinicians and clinical trialists to estimate the probability of an infant’s PDA closing and the chronological age at which closure will likely occur.

#### Aim 2

To determine in preterm infants with PDA within the first postnatal month which echocardiographic predictors and biomarkers are predictive of mortality and severity of respiratory illness at 36-weeks postmenstrual age.

After controlling for respiratory severity and other clinical severity of illness markers (Table 1), we will incorporate longitudinally-measured echocardiographic and biomarker variables from the first 4-weeks postnatal to estimate the specific contribution of PDA to mortality or the need for supplemental respiratory support (oxygen or positive-pressure ventilation) at 36-weeks PMA (*primary outcome*) (Table 3). Such composite primary outcomes are standard in neonatal clinical trials due to competing risks between mortality and other important neonatal outcomes [155, 156].

**Table 3:** Aim 2 Outcome Measures and Mediators at 36-weeks postmenstrual age

Primary Outcome	Secondary Outcomes	Covariates of Interest
Mortality or supplemental oxygen or positive pressure respiratory support at 36-weeks postmenstrual age (*binary*)	*Secondary:* Mortality (*binary*) *Exploratory:* Cardiac performance measures (*binary*) Time to full enteral feeds (*binary*) Oral feeding status (*binary)* Oxygen Dependency (Moderate BPD) (*binary*) Positive-Pressure Dependency (Severe BPD) (*binary*)	Spontaneous PDA closure (*binary*) PDA Duration (*continuous*)

*BPD* bronchopulmonary dysplasia, *PDA* patent ductus arteriosus

An infant’s daily respiratory support modality record at 36-weeks PMA closely correlates with important long-term respiratory and neurodevelopmental outcomes [84, 157]. CLD severity at 36-weeks PMA is the neonatal CLD outcome marker most commonly used in randomized trials [155, 158]. Per the National Institutes of Health Consensus Definition, respiratory support via supplemental oxygen at 36-weeks PMA defines moderate CLD (BPD), whereas invasive or noninvasive ventilation (i.e., continuous positive airway pressure) defines severe CLD (BPD). Infants in the NCH Neonatal Network are never discharged to home prior to 36-weeks PMA unless weaned from all respiratory support including supplemental oxygen (i.e., negative CLD outcome). For those infants transferred before 36-weeks PMA, we will maintain contact with the receiving NICU. In the unlikely case that a transferred infant is lost to follow-up, we will adapt the method of imputing CLD at the time of transfer used by the NICHD NRN to create a validated BPD prediction tool [46, 81].

##### Expected Outcomes

We expect to accurately predict the probability of mortality or requirement for supplemental oxygen or positive-pressure support at 36-weeks PMA using a combination of echocardiographic measures, biomarkers, and clinical measures from the first 4-weeks postnatal. We will develop a clinically useful prediction model that will allow clinicians and those conducting clinical trials to estimate by week in the first 4 postnatal weeks, the probability of mortality or moderate/severe CLD (BPD) development. We will also evaluate the impact of spontaneous PDA closure and duration of PDA exposure on the 36-weeks PMA outcomes. Early prediction of PDA-associated complications will be crucial to future trial development to determine the population of infants who may require interventional PDA treatment and the ideal timing for such an intervention.

#### Aim 3

To determine which echocardiographic predictors and biomarkers in preterm infants with PDA within the first postnatal month are associated with 22 to 26-month neurodevelopmental outcomes.

The extent to which PDA persistence and/or severity contribute to childhood neurodevelopmental impairment is not fully known. The high-risk subset of children who undergo surgical PDA ligation also have the highest probability of neurodevelopmental impairment. Prior to ligation, they have altered cerebral perfusion [11, 159, 160] and long-term decreased cerebral oxygen with associated lower cerebral volumes at term adjusted age relative to infants with earlier PDA closure [11]. For most of the last decade, surgical ligation itself was thought to contribute to worse outcomes [161, 162]. However, a recently published study showed that underlying exposure to severe PDA is a likely cause of worse outcomes [163].

We will incorporate longitudinally-measured echocardiographic and serum biomarker variables from the first 4-weeks postnatal to estimate the specific contribution of PDA to composite motor score (*primary outcome*) and gross motor, fine motor, cognitive composite, and language composite scores (*secondary outcomes*) at 22 to 26-months corrected age (=age since birth–number of weeks born before 40-weeks gestation) (Table 4) as measured by the Bayley Scales of Infant and Toddler Development, 3rd Edition (Bayley III) [164]. The Barratt Simplified Measure of Social Status [165] will be administered to all mothers at study consent to allow adjustment for the influence of socioeconomic status and education within the home environment. Our focus will be on combined motor outcomes at 22 to 26-months because postnatal motor outcomes are nearly unaffected by post-discharge socioeconomic influences [166]. In addition, motor outcomes are a good surrogate for disease-induced global neurologic impairment, because these are largely explained by various degrees of brain injury, whether identified by neuroimaging, or occult and inferred from documented accumulated inflammatory and oxidative stress exposures [167–169].

**Table 4:** Aim 3 Outcome Measures at 22 to 26-months corrected age

Primary Outcome	Secondary and Exploratory Outcomes	Covariates of Interest
Composite Bayley III Motor Score (*continuous*)	*Secondary* Bayley III Gross Motor Development Scaled Standard Score postnatal age (*continuous*) Bayley III Fine Motor Development Scaled Standard Score postnatal age (*continuous*) Bayley III Cognitive Composite Score (*continuous*) Bayley III Language Composite Score (*continuous*) *Exploratory* Supplemental oxygen support (*binary*) Supplemental positive-pressure ventilation support (*binary*) Growth (weight, height, Body Mass Index) (*binary*) Feeding (full oral feeding, gastric-tube) (*binary*)	Spontaneous PDA closure (*binary*) PDA Duration (*continuous*) Oxygen Dependency at 36-weeks postmenstrual age (*binary*)

*Bayley III* Bayley Scales of Infant and Toddler Development, 3^rd^ Edition, *PDA* patent ductus arteriosus

The Bayley III [164] is validated for 22 to 26-months corrected age neurodevelopmental measurement in extremely preterm neonates and is the gold-standard for the evaluation of former NICU graduates [170]. Bayley III scores are normed against a large population and designed to be adjusted for prematurity. Neurodevelopment measurement at 22 to 26-months is standard for major neonatal trial groups including the NICHD NRN [171]. We have demonstrated our ability, as a NICHD NRN study center since 2011, to accurately and prospectively collect 36-week BPD severity data and 2-year neurodevelopmental assessments including Bayley III scores for infants born at 22 to 27-weeks gestational age and/or birth weight <1000 grams in our follow-up programs [172, 173]. The Bayley III is administered in the NCH Neonatal Follow-Up Program by trained examiners who undergo yearly retraining and certification by NRN gold-standards with research follow-up rates at 22 to 26-months exceeding 90%.

Research personnel will work with families to schedule visits at times that best meet family needs. Loss to follow-up will be minimized by rescheduling the Bayley III should a child not be able to complete testing during the originally scheduled time. If necessary, experienced testers will perform the Bayley III in the home environment, as successfully implemented in local NRN studies. All examiners will be masked to randomization group.

We will develop prediction models that will estimate an infant’s probability of motor delays at 22 to 26-months (*primary outcome*). In addition, we will examine the effect of PDA duration as a specific feature of 22 to 26-month neurodevelopmental impairment to more thoroughly evaluate the contribution of extended PDA exposure to these outcomes (Table 4).

##### Expected Outcomes

A combination of echocardiographic measures, biomarkers, and clinical measures collected in the first postnatal month is expected to accurately predict the probability of PDA-associated motor impairment at 22 to 26-months corrected age. A clinically useful prediction model will allow clinicians and those performing clinical trials to estimate, per week within the first postnatal month, the probability of PDA-associated neurodevelopmental impairment at 22 to 26-months corrected age. We will also evaluate the impact of spontaneous PDA closure and duration of PDA exposure on motor outcomes at 22 to 26-months.

### Development of Online Clinical Prediction Tool

PDA treatment parameters and frequencies vary markedly between institutions and individual care providers [17–20]. Over the past decade, the American Academy of Pediatrics [3] has increasingly advocated for a validated PDA-severity grading-system to permit selective treatment of preterm infants with PDA who are most likely to benefit. We will use our final, validated models to create a web-based tool that predicts an infant’s unique probability of spontaneous PDA closure, mortality or 36-weeks PMA respiratory support requirement, and 2-year neurodevelopmental impairment.

### Statistical analyses

#### Aim 1: Statistical Methods

Recognizing that some babies may die prior to PDA closure, models considered will accommodate the competing risk of death prior to closure through a subdistribution hazards model of Fine and Gray [174, 175]. In development of a model to establish a rigorous prediction of PDA closure risk, a penalized variable selection method developed for the Fine and Gray framework will be considered in the model development stage (detailed below) [176]. As a comparison to these models, not accounting for the potential of competing risks will be explored using the Cox model framework. If catheter-based closure is performed (<3% of infants within our regional network), we will censor the observed follow-up time at the time of last recorded PDA, and model the impact of recorded duration of PDA exposure (in postnatal weeks).

##### Model development/training stage

A multivariable prediction model for PDA closure risk will be developed in the training cohort (n=450) based on verified features identified by previous work and our hypothesized new covariates of interest (echocardiographic and serum biomarker measures and magnitude of respiratory support in the first 4-weeks postnatal). The test/validation set that will be recruited in the year following the initial cohort is expected to consist of 225 neonates. Hierarchical clustering will be used to display the unique patterns of PDA closure based on the top echocardiographic, biomarker, demographic, and clinical features. We hypothesize that in addition to echocardiographic and serum biomarker measures, the magnitude of respiratory support in the first 4-weeks postnatal is an important predictor of PDA closure. Higher respiratory support may predict delayed closure and modify the effect of echocardiographic and serum biomarker measures of PDA intensity on closure probability. Therefore, we will examine interactions between respiratory measures (treatment modality, mean daily FiO_2_, and mean oxygen saturation index on the day of the weekly echo) and PDA, in addition to individual features.

A ten-fold cross validation procedure will be iterated 500 times to develop the optimal diagnostic model. In each cross validation iteration, the full training cohort will be randomly split into ten equal partitions/folds. Each fold will take turns to be the test set and the other nine to be the training set. For each fold, a penalized Fine and Gray model regression (e.g., LASSO or SCAD) will be used to select the top predictors of PDA closure risk. The “inner-run” cross-validation and the area under the receiver operating curve (AUC) will be used to choose the optimal tuning parameter. Based on the optimal tuning parameter, the selected model prediction accuracy and sensitivity/specificity will be evaluated based on the test set. An overall receiver operating curve and AUC based on all test set samples will be plotted and estimated after running all 10 folds. The aforementioned cross validation procedure will be replicated 500 times and the distribution of the overall model performance (AUC, specificity, sensitivity) will be obtained. If the average AUC is acceptable (≥0.70) and the variance is limited (0.05), the final model will be estimated with the entire test set.

##### Model validation stage

The final predictive model formulated at the *training stage* will be validated in an independent validation, derived from approximately 225 participants in the cohort recruited the year following enrollment of the initial training cohort. Model prediction accuracy will be evaluated by AUC, with an AUC performance ≥0.70 of interest for further exploration in larger cohorts.

#### Aim 2: Statistical Modeling and Methods

As in Aim 1, we will focus on building predictive models. For the composite outcome of mortality or supplemental oxygen or positive-pressure respiratory support at 36-weeks PMA, we will adopt the same structure as in Aim1, by utilizing the training (450 patients) and validation cohort, recruited thereafter.

Together with clinical features that include respiratory severity and other markers of clinical severity of illness (Table 1), longitudinally-measured echocardiographic and biomarker variables from the first 4-weeks postnatal will be incorporated to predict the composite endpoint of mortality or need for oxygen or positive-pressure support at 36-weeks PMA (*primary outcome*) or mortality (*secondary outcome*). A ten-fold cross validation procedure will be iterated 500 times to develop the optimal diagnostic model. Penalized logistic regression (SCAD, MCP, LASSO) will be utilized to predict our composite outcome and select the top predictors of mortality and CLD risk. We will choose the optimal tuning parameter, determine prediction accuracy and sensitivity/specificity, and estimate the final model with the entire test set as described above.

Given our interest in both the impact of PDA duration and the additional benefit of PDA closure on outcomes (in addition to echo and biomarker predictors over clinical risk factors alone), we will force these two measures (PDA duration and closure) into the model and subsequently compare model fit and accuracy to the models of best predictors that exclude them. Furthermore, we will explore interactions/effect modification between respiratory and other risk factors and PDA measurements (echocardiographic, biomarker) to predict outcomes. Final models (one each for *primary* and *secondary* outcomes) will be fit to the validation cohort, and the model fit summarized as previously detailed.

#### Aim 3: Statistical Analysis

To predict longer-term neurodevelopment outcomes, we will follow the methods described above, utilizing cross validation together with penalized regression models using re-sampling to evaluate the best model choice and to facilitate model building on the training set. As detailed above, we will force PDA measures into our models and evaluate these against models without these measures. For continuous outcomes we will examine the mean square error and Akaike information criterion. Final models for each outcome will be fit and evaluated in the validation cohort. Although we do not expect a large number of patients to be lost to follow-up or to die during follow-up (~10%), we will also explore models that will jointly model neurodevelopment outcomes and time to loss or death with a Cox survival model. We will follow the same general framework for model development as previously described, using a joint random-intercept Cox model [177].

### Sample size and power

Sample size in the training/model building stage is based on the justification according to Dobbin et al. [178] and Pang et al. [179] Given that we expect the majority of PDA closures by 36-weeks PMA (**Aim 1**) following enrollment (upwards of 80-85% of the 450 in the training stage), and the relatively limited set of potential covariates, we expect our resulting classification from the training set to have a tolerance/accuracy of at most 0.05 of the optimal accuracy. An accuracy of 0.05 can be interpreted as the expected accuracy of the classifier to be within 5% of the "best" possible accuracy achieved with a binary classifier. Moreover, with 10-fold cross validation, we expect that power will be high (over 80%) with two-sided type I error of 5% with the expected 450 patient target accrual and modest hazard ratios between 1.5-2.0.

Assuming approximately ¼ of patients (n=112) will experience the **Aim 2** primary outcome event, we expect our resulting classification from the training set to have a tolerance/accuracy of at most 0.05 of the optimal accuracy using the justification according to Dobbin et al. [178, 179]

### Missing Data

We do not anticipate missing covariate data since we will prospectively record data. However, should substantial covariate data be missing, we will employ multiple imputation techniques.

## Discussion

The strengths of this investigation include a prospective cohort design within a unique population of untreated infants with PDA, and innovative modeling using advanced echocardiography, biomarkers, and clinical measures to accurately predict not only spontaneous PDA closure timing, but which infants with persistent PDA are at the highest risk for worsened PDA-associated outcomes (mortality, CLD, neurodevelopmental impairment). This novel approach will fulfill a critical American Academy of Pediatrics clinical research objective [3] by determining the relative added contribution of echocardiography and biomarkers at specific weekly intervals over measurable clinical risk factors alone. Following optimization of our prediction models using 10-fold cross validation and penalized regression, all prediction models will be independently validated in a second cohort of patients.

The results from this study will immediately inform clinical decision-making and, as a critical step to designing trials that will enroll appropriate participants in randomized trials, will ultimately lead to improved outcomes for preterm infants with PDA. Our weekly prediction models for the first postnatal month, the period when pharmacologic and early interventional closure decisions must be made, will aid practicing clinicians to avoid pharmacologically treating infants unlikely to benefit, and randomized-trial planners to develop evidence-based trial designs to determine whether pharmacologic and interventional PDA closure is beneficial in specific prenatal infant populations, and when PDA treatment is most effective. Because of our diverse population of <30-weeks gestation preterm infants within both delivery and referral hospitals’ NICUs in one of the largest allied NICU networks in the United States, we anticipate findings will be applicable to similar preterm infants with PDA throughout the U.S. and developed world.

Although this study uses a prospective cohort and rigorously designed observational study design to answer proposed questions that cannot be answered with a randomized trial, but that are crucial to future PDA trial development, confounding is a potential problem [3]. We will carefully control for known confounders using clinical markers that are validated predictors of our primary outcomes.

Early mortality precludes PDA-closure and development of PDA-associated outcomes. Our team has experience in applying composite outcomes and guarding against mortality-associated biases, such as for immortal-time bias, in the preterm infant population [18, 180]. For **Aim 2** we will employ a primary mortality or CLD composite outcome at 36-weeks PMA, the accepted practice among neonatal randomized trialists. For **Aim 1** (spontaneous closure) outcomes we will employ Fine and Gray subdistribution hazards models, which will allow us to account for competing mortality risks and limit the analysis to observed follow-up time. In **Aim 3** (neurodevelopmental outcomes at 22 to 26-months) we will explore using joint models to account for drop out and potential death.

## Supplementary information

**Additional file 1: Table S1.** World Health Organization Trial Registration Data Set. (PDF 94 kb)

## Data Availability

Data sharing is generally not applicable to this publication as no datasets outside of the presented pilot data were generated or analysed specifically for this prospective cohort protocol. We will share our deidentified pilot data upon request to the corresponding author (CB) and will welcome the opportunity to share data in the future pending the completion of our investigation. Following the completion and publication of our investigation, all patient identifiers will be removed from the final dataset and we will make the data available to other users under a data sharing agreement. We will ask under the agreement that other users will commit to using the data only for research purposes, that they will not identify individual study subjects, that they will securely store the data electronically using password protection or encryption, that they will delete or destroy the data after the completion of their investigation, and that they will acknowledge the contribution of the funding agency (NIH NHLBI) and our investigative team in collecting the original data. Additional study details are summarized in Additional file 1: Table S1.

## Abbreviations

AUC: Area under the receiver operating curve
Bayley III: Bayley Scales of Infant Development, 3^rd^ Edition
BPD: Bronchopulmonary dysplasia
CLD: Chronic lung disease
FiO_2_: Fraction of inspired oxygen
NCH: Nationwide Children’s Hospital
NICHD: *Eunice Kennedy Shriver* National Institute of Child Health and Human Development
NICU: Neonatal intensive care unit
NRN: NICHD Neonatal Research Network
PDA: Patent ductus arteriosus
PMA: Postmenstrual age
