# Supplementary material for: Early prediction of spontaneous Patent Ductus Arteriosus (PDA) closure and PDA-associated outcomes: a prospective cohort investigation
Source: BMC Pediatr. 2019 Sep 13;19:333. doi: 10.1186/s12887-019-1708-z (PMC6743099; doi:10.1186/s12887-019-1708-z)
Supplement: Supplementary file 1 — Additional file 1: Table S1. World Health Organization Trial Registration Data Set. (PDF 94 kb) [file 12887_2019_1708_MOESM1_ESM.pdf]

Supplementary Appendix

Early Prediction of Spontaneous Patent Ductus Arteriosus (PDA) Closure and PDA-Associated Outcomes: A Prospective Cohort Investigation

JL Slaughter, CL Cua, JL Notestine, BK Rivera, L Marzec, EM Hade, NL Maitre, MA Klebanoff, M Ilgenfritz, VT Le, DJ Lewandowski, CH Backes

Supplemental Table 1. World Health Organization Trial Registration Data Set

Data category	Information
Primary registry and trial identifying number	ClinicalTrials.gov<br>NCT03782610
Date of registration in primary registry	20 December, 2018
Secondary identifying numbers	IRB18-00684, 1800684, R01HL145032
Source(s) of monetary or material support	National Heart, Lung, and Blood Institute of the National Institutes of Health (U.S.)
Primary sponsor	National Heart, Lung, and Blood Institute
Secondary sponsor(s)	Not Applicable
Contact for public queries	CB, MD [email address]
Contact for scientific queries	CB, MD Department of Pediatrics, Nationwide Children’s Hospital, Columbus, Ohio, USA
Public title	Patent Ductus Arteriosus (PDA) Prediction Study
Scientific title	Early Prediction of Spontaneous Patent Ductus Arteriosus (PDA) Closure and PDA-Associated Outcomes
Countries of recruitment	United States
Health condition(s) or problem(s) studied	Patent ductus arteriosus
Intervention(s)	Not Applicable
Key inclusion and exclusion criteria	Ages eligible for study: up to 72 hours (child); Sexes eligible for study: all; Accepts healthy volunteers: no<br>Inclusion criteria:<br>Infants born between 23-weeks + 0 days ($23^{0/7}$ weeks) and $29^{6/7}$ weeks of gestation, inclusive;<br>Admitted to a study network neonatal intensive care unit within 72-hours of birth;<br>PDA noted on initial screening echocardiogram at <72 postnatal hours

	Exclusion criteria:<br>Life-threatening congenital abnormalities including congenital heart disease (other than PDA or small atrial septal defects/patent foramen ovale/muscular ventriculoseptal defects);<br>Infants whose parents have chosen to allow natural death (do not resuscitate order)
Study type	Observational (Patient Registry)<br>Observational model: Cohort<br>Primary purpose: Estimate probability of spontaneous PDA closure and predict the timing of ductal closure using clinical and echocardiographic predictors within the first postnatal month.
Date of first enrolment	April 2019
Target sample size	675
Recruitment status	Recruiting
Primary outcome(s)	1. PDA closure documented via echo by 36-weeks PMA (binary) [Time Frame: Outcome will be documented between <72-hour screening echo and 36-weeks PMA.]<br>2. Mortality or supplemental oxygen or positive-pressure respiratory support at 36-weeks PMA (binary) [Time Frame: Outcome will be documented between <72-hour screening echo and 36-weeks PMA]<br>3. Composite Bayley III Motor Score at 22-26 months CA (continuous). [Time Frame: Bayley III Score Testing will occur at 22 to 26-months CA]
Key secondary outcomes	1. Mortality by 36-weeks PMA (binary) [Time Frame: Death occurring between 72-hours postnatal and 36-weeks PMA]<br>2. Bayley III Gross Motor Development Scaled Standard Score at 22-26 months CA (continuous) [Time Frame: Recorded at 22-26 months CA]<br>3. Bayley III Fine Motor Development Scaled Standard Score postnatal age at 22-26 months CA (continuous) [Time Frame: Recorded at 22-26 months CA]<br>4. Bayley III Cognitive Composite Score at 22-26 months CA (continuous) [Time Frame: Recorded at 22-26 months CA]<br>5. Bayley III Language Composite Score at 22-26 months CA (continuous) [Time Frame: Recorded at 22-26 months CA]

Bayley III, Bayley Scales of Infant and Toddler Development 3rd Edition; CA, corrected age (age since birth - number of weeks born before 40-weeks gestation); PDA, patent ductus arteriosus; PMA, postmenstrual age
